# CHILDREN’S RESIDENTIAL PROXIMITY, SPOUSAL PRESENCE, AND MODIFIABLE RISK FACTORS FOR DEMENTIA

**DOI:** 10.1093/geroni/igae098.1294

**Published:** 2024-12-31

**Authors:** Zhuoer Lin, Xuecheng Yin, Becca Levy, Yue Yuan, Xi Chen

**Affiliations:** University of Illinois Chicago, Chicago, Illinois, United States; Oklahoma State University, Stillwater, Oklahoma, United States; Yale University School of Public Health, New Haven, Connecticut, United States; Lehigh University, Bethlehem, Pennsylvania, United States; Yale University, New Haven, Connecticut, United States

## Abstract

**Objectives:**

Cognitive impairment poses considerable challenges among older adults, with the role of family support becoming increasingly crucial. This study examines the association of children’s residential proximity and spousal presence with key modifiable risk factors for dementia in cognitively impaired older adults.

**Methods:**

We analyzed 14,731 persons (35,840 person-waves) aged 50 and older with cognitive impairment from the Health and Retirement Study (1995-2018). Family support was categorized by spousal presence and children’s residential proximity. Modifiable risk factors, including smoking, depressive symptoms, and social isolation, were assessed. Associations between family support and the modifiable risk factors were determined using mixed-effects logistic regressions.

**Results:**

A significant proportion of older adults with cognitive impairment lacked access to family support, with either no spouse (47.0%) or all children living over 10 miles away (25.2%). Those with less available family support, characterized by distant-residing children and the absence of a spouse, had a significantly higher prevalence of smoking, depressive symptoms, and social isolation. Moreover, we revealed a consistent gradient in the prevalence of the risk factors by the degree of family support. Relative to older adults with a spouse and co-resident children, those without a spouse and with all children residing further than 10 miles displayed the highest prevalence of the risk factors. These findings were robust to various sensitivity analyses. Conclusions: Family support from spouses and nearby children serves as a protective factor against modifiable dementia risk factors in cognitively impaired older adults. Strengthening family and social support is paramount for this vulnerable population.

